# Bronchial stump reinforcement with an azygous vein flap

**DOI:** 10.1186/1749-8090-4-22

**Published:** 2009-05-28

**Authors:** Faisal Al-Mufarrej, Marc Margolis, Eric Strother, Barbara Tempesta, Farid Gharagozloo

**Affiliations:** 1 The George Washington University Medical Center, Department of Surgery, 2300 Eye Street NW, Washington, DC 20037, USA; 2 Washington Institute of Thoracic and Cardiovascular Surgery, 2175 K Street NW, Washington DC 20037, USA; 3 Department of Surgery, Division of Cardio-Thoracic Surgery George Washington Medical Faculty Associates 2150 Pennsylvania Avenue, NW, Washington, DC 20037 USA

## Abstract

Bronchial stump reinforcement has been shown to significantly reduce the incidence of bronchopleural fistulas. Various coverage techniques have been described in the literature. While the azygous vein flap is an easy, safe and effective reinforcement option for right-sided bronchial stumps, the flap is not widely adopted, with little mention in the literature, partly due to surgeons' uneasiness with the technique. In this report, we describe an easy-to-adopt approach to azygous vein bronchial reinforcement.

## Introduction

A bronchopleural fistula is a serious complication of lobectomies and, more commonly, pneumonectomies. It can lead to persistent empyemas that require open drainage, frequent debridements, and prolonged hospitalization. While many patient, disease and technical risk factors for the development of bronchopleural fistulas have been identified, bronchial stump reinforcement has been shown to significantly reduce the incidence of this complication. Various coverage techniques have been described in the literature, with intercostal, diaphragmatic, and pericardial flaps being the most widely studied. While the azygous vein flap is an easy, safe and effective reinforcement option for right-sided bronchial stumps, the flap is not widely adopted, with little mention in the literature, partly due to surgeons' uneasiness with the technique. In this report, we describe an easy-to-adopt approach to azygous vein bronchial reinforcement.

## Technique

**Figure 1:**
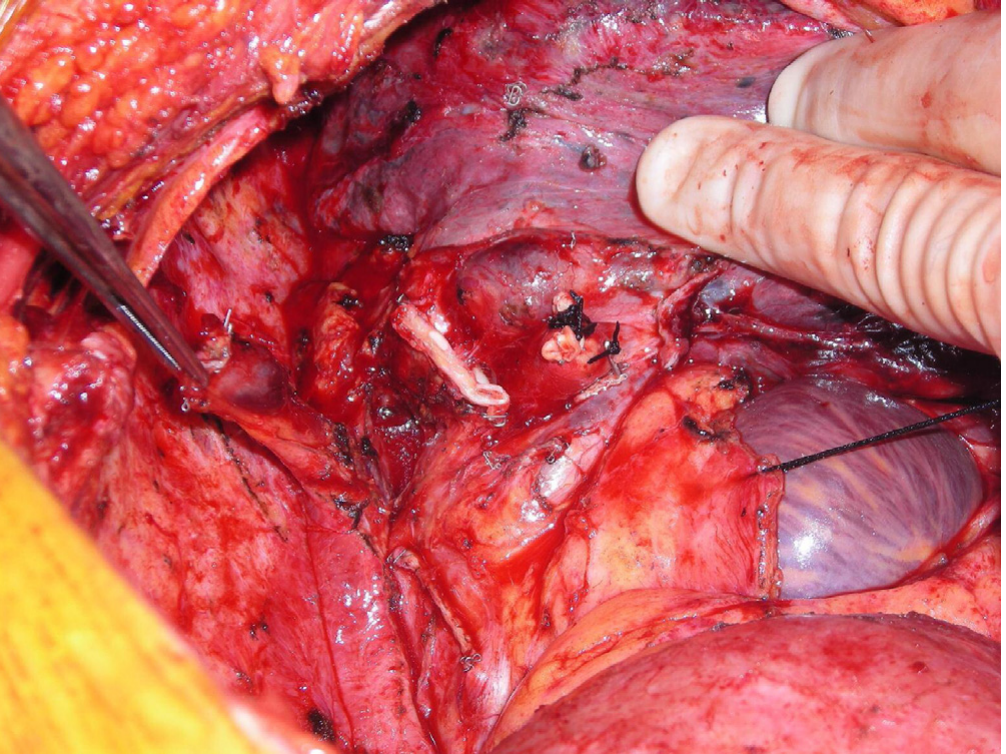
**Dissection, distal stapling/division, and mobilization of azygous vein**.

**Figure 2:**
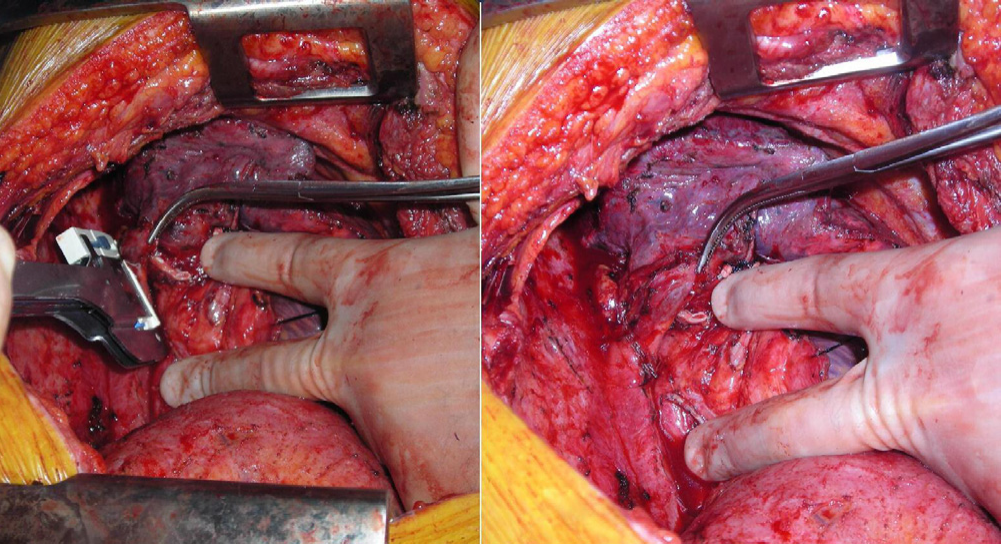
**Proximal stapling of azygous vein**.

**Figure 3:**
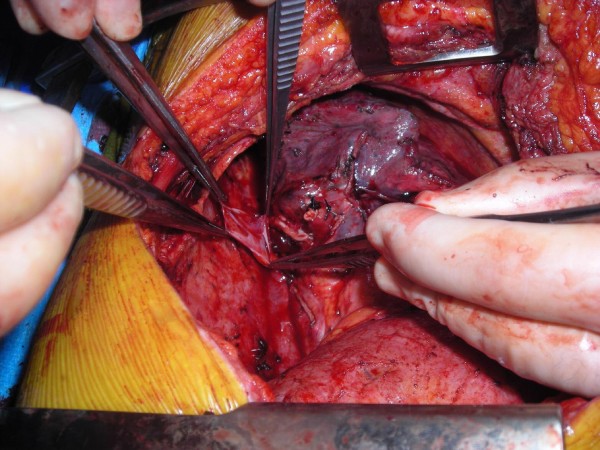
**Posterior, longitudinal opening of distal vein and creation of flap**.

**Figure 4:**
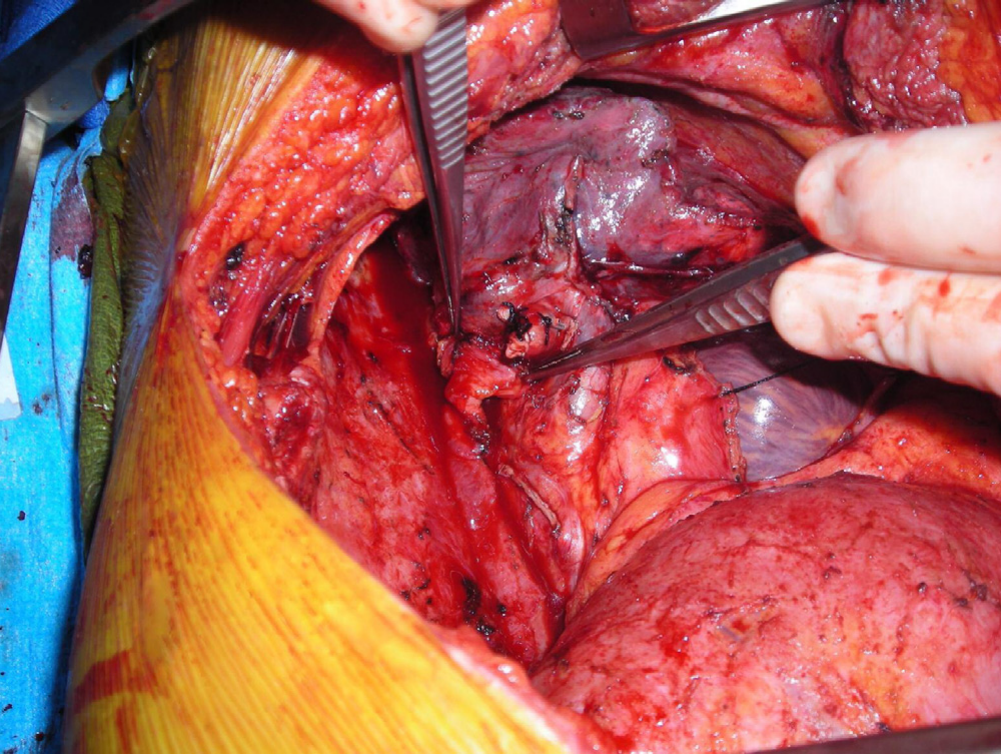
**Securing the azygous vein flap to the bronchial stump**.

Upon completing a right-sided, high-risk lobectomy or a right pneumonectomy, the azygous vein is dissected off the posterior chest wall between the point where the right superior intercostal vein drains into it and where it drains into the superior vena cava. The right superior intercostal vein (draining the second, third, and fourth posterior intercostal veins) is then ligated and divided. With the aid of a vascular endoscopic gastrointestinal anastamosis (Endo-GIA Roticulator 45) stapler, the azygous vein is stapled and divided close to where it drains into the superior vena cava. The azygous vein is then mobilized until enough length is obtained (Figure 1). The fifth and, rarely, subsequent posterior intercostal veins may need to be ligated and divided to gain some length on the azygous flap. Using a linear stapler (TA-30), the azygous vein is stapled across its most proximal end (Figure 2). The posterior aspect of the azygous vein, between the two staple lines, is then opened up longitudinally, creating a flap that can cover the stapled or sutured bronchial stump (Figure 3). Finally, the flap is secured over the bronchial stump with simple, interrupted No. 0000 PDS sutures (Figure 4).

## Discussion

A post-pneumonectomy or post-lobectomy bronchial stump fistula is a serious complication with a reported incidence between 0 and 12% [1]. Several risk factors for the development of bronchopleural fistulas have been identified. The incidence is higher after surgery for benign disease and after pneumonectomies (especially right-sided ones). A fistula is more likely with lower pre-operative FEV1 and DLco, with increased intravenous fluid in the first 12 hours, and with blood transfusions. Bronchial stump closure with staples seems to be protective against bronchopleural fistulas when compared with suture closure [2].

Furthermore, studies suggest that bronchial stump reinforcement with viable tissue is prophylactic against the development of bronchopleural fistulas [2,3]. Because the stump closes by callous formation on its serosal surface, the flap greatly accelerates healing. Various coverage techniques have been described in the literature. Intercostal and diaphragmatic muscle flaps are probably the two best described stump reinforcement techniques [1,3,4]. While stump reinforcement may protect against bronchopleural fistulas, the use of muscle flap reinforcement seems to increase blood loss, chest-tube volume, and pain score after surgery when compared to conventional pneumonectomies [5]. Comparing the various muscle flap techniques, one study suggested that intercostal muscle flaps may be associated with a smaller peri-operative morbidity than diaphragmatic flaps, although the difference was not statistically significant [4]. Diaphragmatic flaps may also be associated with the development of diaphragmatic hernias [4]. The diaphragmatic muscle flap, however, has the advantage of length, pliability and consistent vascular anatomy. They are also thought to not adversely affect pulmonary function. Pedicled full-thickness diaphragmatic flaps are a good option in cases of extended pneumonectomy with pericardial resection that require not only stump coverage but also pericardial defect closure [4].

Pericardial coverage of the bronchial stump is another option. It can be achieved with either a portion of posterior pericardium or with a generous pedicled pericardial flap (and concomitant reconstruction of the pericardium with mesh) [1]. Pericardial flaps, however, are associated with the development of post-operative pericarditis, and in cases of large pedicled flaps, they may be associated with pericardial tamponade if the reconstruction is made too tight. Moreover, in patients with extensive malignant disease, the pericardium may be involved and not a good stump reinforcement option. While pleural [1] and mediastinal fat reinforcements [4] seem to offer protection against the development of bronchopleural fistulas, they seem to be slightly less effective than pericardial, intercostal, and diaphragmatic flaps [1,5].

With regards to azygous vein flaps, studies have proven them to be effective [1], and, clearly, as described above, this reinforcement technique is easy and quick with little associated risks. While it is only applicable to right-sided bronchial stumps, this technique also allows for the preservation of the intercostals and the diaphragm for the treatment of any possible bronchopleural fistula in the future, obviating the need for an omental or an extra-thoracic muscle flap closure in those cases. Despite all these advantages, the technique has not been widely adopted and has little mention in the current literature. However, surgeons should become as familiar with this technique as they are with the other more common reinforcement techniques.

## Competing interests

The authors declare that they have no competing interests.

## Authors' contributions

FM, MM, ES, BT and FG were all involved in the preparation of this manuscript including the background literature search and the photography. All authors read and approved the final manuscript.
